# Gudder’s Theorem and the Born Rule

**DOI:** 10.3390/e20030158

**Published:** 2018-03-02

**Authors:** Francisco De Zela

**Affiliations:** Departamento de Ciencias, Sección Física, Pontificia Universidad Católica del Perú, Apartado 1761, Lima, Peru; fdezela@pucp.edu.pe

**Keywords:** Born probability rule, quantum-classical relationship, spinors in quantum and classical physics, 03.65.Ta, 03.65.Ud, 02.50.Ey, 42.50.Xa

## Abstract

We derive the Born probability rule from Gudder’s theorem—a theorem that addresses orthogonally-additive functions. These functions are shown to be tightly connected to the functions that enter the definition of a signed measure. By imposing some additional requirements besides orthogonal additivity, the addressed functions are proved to be linear, so they can be given in terms of an inner product. By further restricting them to act on projectors, Gudder’s functions are proved to act as probability measures obeying Born’s rule. The procedure does not invoke any property that fully lies within the quantum framework, so Born’s rule is shown to apply within both the classical and the quantum domains.

## 1. Introduction

Originally, Born’s probability rule was considered to be one of those salient features of quantum theory which make it markedly depart from a classical description of physical phenomena. Born’s rule was complemented by another one, which is a prescription that establishes how a system changes when submitted to measurement: the so-called collapse rule. There have been some attempts to derive the Born rule from basic concepts of probability theory, thereby reducing the axiomatic basis of quantum mechanics. Notably, Gleason’s theorem [1] claims to achieve such a reduction by deriving the Born rule from the properties of a probability measure. However, Gleason’s theorem does not hold for two-dimensional quantum systems (i.e., for qubits). This is also the case with a prominent corollary of Gleason’s theorem, the Bell–Kochen–Specker (BKS) theorem [2,3], which disproves the assumption that it is always possible to assign noncontextual values to observables prior to measurement. Thus, in the quantum framework, it is not possible to interpret measurement outcomes as revealing pre-existing values of the measured observables. However, such a fundamental claim does not include qubits. Moreover, Bell violations showing the impossibility of hidden-variable models require composite systems [2,4]. It is thus possible to construct a hidden-variable model for a single qubit [3,5]. This state of affairs has prompted some people to place qubits—and them alone—into a sort of limbo, as being half quantum and half classical objects [6,7]. Indeed, as pointed out in [8], it is widely believed that “a single qubit is not a truly quantum system”. No matter how appealing the motivations for such a belief might seem, its untenability becomes clear when seen from the perspective of the quantum formalism alone: there is nothing in this formalism that distinguishes two-level systems from other systems of higher dimensionality. We should therefore simply admit that Gleason’s approach does not meet its intended goal.

The inclusion of qubits was achieved in Busch’s extension [9] of Gleason’s theorem. Instead of the pairwise orthogonal projectors $P_{i}$ entering Gleason’s theorem, Busch addresses positive operator-valued measures (POVMs) $E_{n}$. However, the inclusion of qubits in Busch’s approach was obtained at the cost of departing from our most intuitive notion of a measure. The mathematical tool that corresponds to our basic notion of a measure is a non-negative function *m* over a σ-algebra. This function is required to satisfy m(A∪B)=m(A)+m(B), whenever A∩B=∅. The last condition must hold because in case A∩B≠∅, we should subtract m(A∩B) from m(A)+m(B) in order to encompass our intuitive notion of a measure. A particular and important case is the “probability measure”. In quantum mechanics, this measure is defined over the projection lattice P(H) of a Hilbert space H, and it is thus consistent to require for $P_{i},P_{j}\in P\left(H\right)$ that $m\left(P_{i}+P_{j}\right)=m\left(P_{i}\right)+m\left(P_{j}\right)$, whenever $P_{i}P_{j}=0$. On the other hand, it is rather unnatural to call *v* a measure if it is required to satisfy $v\left(E_{n}+E_{m}\right)=v\left(E_{n}\right)+v\left(E_{m}\right)$, even though $E_{n}E_{m}\neq 0$. However, this is the case in Busch’s extension of Gleason’s theorem, in which projectors are replaced by POVMs. As for the BKS theorem, Cabello [8] has similarly proved its validity in the case of qubits by replacing projective measurements with POVMs, while Aravind [10] extended Cabello’s proof to arbitrary finite dimensions. The introduction of POVMs in the quantum formalism as a generalization of von Neumann’s projection-valued measures has been required for various reasons, such as the quantum information approach to quantum mechanics, the employment of non-optimal devices that deliver unsharp measurement outcomes, the description of composite measurements, etc. However, none of these reasons bears any particular connection with two-state systems. It is thus unclear why the inclusion of qubits in the aforementioned theorems should require the replacement of projective measurements by POVMs.

Recently, we have presented an alternative derivation of the Born rule [11], starting from Gudder’s theorem [12]—a theorem which is in a sense the reciprocal of Pythagoras’s theorem. Such a derivation begins with two-dimensional systems and then extends to higher-dimensional ones, including both pure and mixed states. By observing that the Born rule involves only two states, its derivation can be generally reduced to the two-dimensional case, irrespective of the (finite or infinite) dimensionality of the addressed vector space. Moreover, the derivation blurs the distinction between quantum and classical measurements, so Born’s rule is shown to apply beyond its original purely quantum domain. This opens the way for the construction of hidden-variable models of Bell violations produced by maximally entangled states [13].

Hall [14] recently criticized our derivation of the Born rule, arguing that a non-linear counterexample that shows why qubits are excluded from the scope of Gleason’s theorem also applies in our approach. One of the purposes of the present work is to show that this is not so. The reason can be stated very simply and in advance: the assumptions underlying our approach imply that any function we deal with is a linear one. This was not explicitly shown in [11], but only implicitly, by deriving Born’s linear expression. We present here an explicit demonstration of linearity, and moreover, go beyond the goals of our previous work. Indeed, Hall’s criticisms represent a welcome opportunity to expand the scope of Ref. [11], as well as to clear up the physical content of the proposed extension of Gleason’s theorem.

We should stress that we do not attempt to solve the so-called “measurement problem”; that is, we do not attempt to answer the question as to how measurements fit into the quantum formalism. Instead, we follow a similar approach as in Ref. [15] and take measurements as something fundamental that require a proper self-consistent description. Thus, we restrict ourselves to the probability rule, leaving aside the collapse rule and the question as to whether collapse is a physical process or just an updating of our system’s knowledge. On the other hand, we do address the question about the placement of the Born rule with respect to the quantum–classical border. To this day, the latter remains a controversial issue [16,17,18,19,20,21,22,23,24], to which the present work intends to make a contribution.

This paper is organized as follows. In Section 2 we recall Gleason’s theorem and in Section 3 we reproduce—for the sake of completeness—the essential points of Ref. [11]. At the same time, we extend somewhat the results presented in Ref. [11], by completely fixing the orthogonally additive function that we addressed there and that was left partially undefined in the cited work. We also address Hall’s criticisms. In Section 4 we present an alternative derivation of the Born rule which bypasses the reduction to two-level systems that was used in Ref. [11], and generally applies to *N*-level systems, with N≥2. We close the paper by discussing our results.

## 2. Gleason’s Theorem and Its Restriction to Dimensions Greater Than Two

Let us recall Gleason’s theorem. It states that any probability measure over the lattice P(H) of orthogonal projectors $P_{i}\in P\left(H\right)$ acting on a Hilbert space H has the form given by the Born rule [1]. The defining properties of a probability measure $m\left(P\right):P\left(H\right)\to \left[0,1\right]$ read as follows:

(1) m(I)=1,

(2) $$m\left(\sum_{i}P_{i}\right)=\sum_{i}m\left(P_{i}\right).$$

It is straightforward to show that $\sum_{i}P_{i}\in P\left(H\right)$ implies that $P_{i}P_{j}=0,$ for i≠j . Gleason proved that whenever dimH≥3, there exists a unique density operator ρ such that

(3) $$m\left(P\right)=\mathrm{Tr}\left(\rho P\right),\;\forall P\in P\left(H\right),$$

which is the Born rule.

The exclusion of qubits from the scope of Gleason’s theorem may be traced back to the fact that assumptions ((1) and (2))—in particular (2)—are not strong enough to imply Equation (3) in the two-dimensional case. Indeed, Gleason’s proof requires showing that *m* is continuous. This can be done only for dimH≥3. In the 2D case, there are discontinuous measures satisfying assumptions ((1) and (2)). While Gleason’s proof is technically difficult (and for this reason the exclusion of the 2D case is not quite transparent), in the case of its prominent corollary, the BKS theorem, it is easier to understand why the latter does not hold in the 2D case. Indeed, an independent demonstration of the BKS theorem—i.e., not as a corollary of Gleason’s—can be reduced to the task of coloring the surface of a unit hyper-sphere with two colors [7]. This is possible for two dimensions—viz., in the case of the unit circle—but not for higher dimensions.

There is yet another way to show that the 2D case must lie outside the domain of Gleason’s theorem. We observe that measure m(P) entering Born’s rule (see Equation (3)) is not only continuous, but also linear. Hall [14] provided a non-linear measure *m* over the set of qubit-projectors which satisfies conditions ((1) and (2)), thereby proving that Gleason’s theorem cannot hold for qubits. As for the derivation of the Born rule that we reported in [11], the conditions we impose on the addressed measures can be satisfied only by linear functions. This notwithstanding, Hall claimed to have provided a non-linear function satisfying said conditions [14]. Below, we will discuss what went wrong in Hall’s reasoning.

## 3. Gudder’s Theorem and the Born Rule for Two-Level Systems

Linearity is a central issue in the derivation of Born’s rule from any chosen assumptions [9,15,25,26,27,28]. For instance, the derivation in Ref. [9]—which includes qubits—entails the demonstration that the measure v(E) over POVMs is a positive linear functional that can be obtained from a density operator. As we have seen, Gleason’s assumptions are instead too weak to enforce linearity in the case of qubits. In our approach, linearity is enforced by imposing upon the concept of a measure a series of requirements that reflect the most general experimental procedures. These requirements generally apply when submitting any system to measurement. As stressed in Ref. [11], our assumptions are not restricted to the quantum case, and therefore some classical measurements can also be encoded in terms of the Born rule. Said assumptions are strongly driven by physical considerations rather than by mathematical motivations.

Most measurement procedures in physics are essentially “counting” procedures. They consist of counting how many times a given unit—a measure—fits into the observable that is submitted to measurement. As already said, the primary standard mathematical tool that captures our basic notion of a measure is a non-negative function *m* over a σ-algebra. The restriction to be non-negative is a convenient one in some cases, such as integration theory. Instead, in physics it is often convenient to distinguish between, e.g., two sides (left and right), or to be able to add and subtract a given amount. Hence, a generalization of the original concept of measure is convenient, to what is called a signed measure μ. A signed measure is defined over a σ-algebra $A_{\sigma }$, as $\mu :A_{\sigma }\to R$, with $\mu \left(\cup_{n}A_{n}\right)=\sum_{n}\mu \left(A_{n}\right)$, for any sequence $A_{1},A_{2},\ldots ,A_{n}$ of pairwise disjoint sets in $A_{\sigma }$. Besides these mathematical requirements, we can include some additional ones that reflect our dealing with physical measurements. First of all, we restrict ourselves to dealing with continuous functions *f*. This requirement captures our basic notion that infinitesimal variations of the observable being measured should lead to infinitesimal variations of the measurement result. Second, we restrict ourselves to dealing with functions *f* that are defined over an inner product vector space *V*. With these restrictions, what was initially a signed measure ends up being the subject matter of Gudder’s theorem [12]. Indeed, Gudder’s theorem deals with an inner product vector space *V* and a continuous function *f* that is orthogonally additive. The definition of such a function reads as follows:

**Definition** **1.** (4) $$f:V\to R\;\mathrm{is}\;\mathrm{orthogonally}\;\mathrm{additive}\;\mathrm{if}\;f\left(r+r^{\prime }\right)=f\left(r\right)+f\left(r^{\prime }\right)\;\;\mathrm{whenever}\;\;r\cdot r^{\prime }=0.$$

Gudder proves that the following result holds true:

**Theorem** **1.** *If f:V→R is orthogonally additive and continuous, then it has the form*

(5) f(r)=c(r·r)+k·r,

*where c∈R and k∈V.*

Our aim is to show how Born’s rule arises from Gudder’s theorem. To this end, we first focus on qubits. A qubit can be represented by a unit vector $|\phi \rangle \in H_{2}$ of an equivalence class—a so-called “ray”—or alternatively, it can be represented by the corresponding projector

(6) $$P_{\phi }\equiv \left|\phi \rangle \langle \phi \right|=\frac{1}{2}\left(I_{2}+{\hat{n}}_{\phi }\cdot \sigma \right).$$

Here, $I_{2}$ is the identity operator in $H_{2}$ and the unit vector ${\hat{n}}_{\phi }=\mathrm{Tr}\left(\sigma P_{\phi }\right)$, with σ standing for the triple of Pauli matrices. In general, for a non-normalized qubit $|\psi \rangle \in H_{2}$, we can write

(7) $$R_{\psi }\equiv \left|\psi \rangle \langle \psi \right|=\frac{1}{2}\sum_{\mu =0}^{3}r_{\mu }\sigma_{\mu },$$

with $\sigma_{0}\equiv I_{2}$ and $r_{\mu }=\mathrm{Tr}\left(\sigma_{\mu }R_{\psi }\right)$. We see that $R_{\psi }=P_{\psi }$ whenever 〈ψ|ψ〉=1. There is a one-to-one correspondence between operators $R_{\psi }$ and vectors $r:=\left(r_{0},r_{1},r_{2},r_{3}\right)\equiv \left(r_{0},r\right)$. The latter span a four-dimensional real vector space $V_{4}$ that can be made an inner product space by defining the Euclidean inner product

(8) $$r\cdot r^{\prime }=\sum_{\mu =0}^{3}r_{\mu }r_{\mu }^{\prime }.$$

We now wish to define a measure $f_{\phi }$ that is associated to a particular qubit $|\phi \rangle ↔r_{\phi }\equiv \left(1,{\hat{n}}_{\phi }\right)$. In a sense, $f_{\phi }$ and |ϕ〉 represent one and the same physical object that is mathematically encoded in two alternative ways [11]. To start with, $f_{\phi }$ must satisfy the following requirements.

(1) $f_{\phi }$ must satisfy the assumptions of Theorem 1.
(2) $f_{\phi }\left(r_{\phi }\right)=1$, which corresponds to requiring that our unit of measure fits exactly one time into itself.
(3) $f_{\phi }\left(r_{\phi_{⊥}}\right)=0$ for the vector $|\phi_{⊥}\rangle ↔r_{\phi_{⊥}}\equiv \left(1,-{\hat{n}}_{\phi }\right)$ that is orthogonal to |ϕ〉.
  On applying Gudder’s theorem with $k=(k_{0},k)$, we obtain
  (9) $$f_{\phi }\left[(1,{\hat{n}}_{\phi })\right]=2c+k_{0}+{\hat{n}}_{\phi }\cdot k=1,$$
  (10) $$f_{\phi }\left[(1,-{\hat{n}}_{\phi })\right]=2c+k_{0}-{\hat{n}}_{\phi }\cdot k=0.$$
  From these equations, we get $2c+k_{0}=1/2$ and ${\hat{n}}_{\phi }\cdot k=1/2$. Up to this point, we have been dealing with a function $f_{\phi }$ that is not necessarily identifiable with a probability measure. Let us further restrict $f_{\phi }$ to satisfy the following requirement:
(4) $f_{\phi }\left[(1,{\hat{n}}_{\psi })\right]\in \left[0,1\right]$ for any four-vector $\left(1,{\hat{n}}_{\psi }\right)↔\left|\psi \rangle \langle \psi \right|=P_{\psi }$.
  In such a case, $f_{\phi }\left[(1,{\hat{n}}_{\psi })\right]=2c+k_{0}+{\hat{n}}_{\psi }\cdot k=1/2+{\hat{n}}_{\psi }\cdot k\in \left[0,1\right]$; i.e.,
  (11) $$-\frac{1}{2}\leq \left|k\right|\cos\theta \leq \frac{1}{2},$$
  where $\cos\theta ={\hat{n}}_{\psi }\cdot \hat{k}$ spans the interval $\left[-1,1\right]$ under variation of ${\hat{n}}_{\psi }$. This implies that |k|=1/2, hence $k={\hat{n}}_{\phi }/2$, and we can finally write
  (12) $$f_{\phi }\left[(1,{\hat{n}}_{\psi })\right]=\frac{1}{2}\left(1+{\hat{n}}_{\phi }\cdot {\hat{n}}_{\psi }\right).$$

Using $P_{\psi }=\left|\psi \rangle \langle \psi \right|=\left(I_{2}+{\hat{n}}_{\psi }\cdot \sigma \right)/2$ and similarly for $P_{\phi }=\left|\phi \rangle \langle \phi \right|$, we can write $f_{\phi }\left(P_{\psi }\right)$ in the standard form

(13) $$f_{\phi }\left(P_{\psi }\right)={\left|\langle \phi |\psi \rangle \right|}^{2}=\mathrm{Tr}\left(P_{\phi }P_{\psi }\right).$$

The measure $f_{\phi }$ we have obtained under the above requirements can be consistently interpreted as a probability measure. We have put our requirements on a function $f_{\phi }$ that applies to vectors $r\in V_{4}$ in general. It is just in order to fix some of the parameters that define $f_{\phi }$ (i.e., *c* and $k=(k_{0},k)$) that we conveniently applied $f_{\phi }$ to some particular vectors $\left(1,\hat{n}\right)\in V_{4}$. These vectors belong to $V_{4}$ in spite of carrying only two independent parameters—the ones fixing $\hat{n}$. Now, as for the function $f_{\phi }$, it has not been completely fixed. Though we know its action on vectors of the form $\left(1,\hat{n}\right)$ (see Equations (9) and (10)), we do not know its action on more general vectors $r\in V_{4}$. This is because we have fixed only $k={\hat{n}}_{\phi }/2$, while *c* and $k_{0}$ remain yet undetermined. In order to fix them, we can consider the vector $\left(-1,{\hat{n}}_{\phi }\right)$, which is orthogonal to $|\phi \rangle ↔r_{\phi }\equiv \left(1,{\hat{n}}_{\phi }\right)$. Thus, we must consistently require that

(14) $$3a)f_{\phi }\left[(-1,{\hat{n}}_{\phi })\right]=2c-k_{0}+{\hat{n}}_{\phi }\cdot k=2c-k_{0}+\frac{1}{2}=0.$$

On account of the above equation and $2c+k_{0}=1/2$, we get c=0 and $k_{0}=1/2$. Hence, $k=r_{\phi }/2$ and Theorem 1 establishes that $f_{\phi }$ is a linear function given by $f_{\phi }\left(r\right)=k\cdot r$; i.e.,

(15) $$f_{\phi }\left[\left(r_{0},r\right)\right]=\frac{1}{2}\left(r_{0}+{\hat{n}}_{\phi }\cdot r\right).$$

On view of $\left(r_{0},r\right)↔R_{\psi }\equiv \rho_{\psi }=\sum_{\mu }r_{\mu }\sigma_{\mu }/2$ (see Equation (7)), and $\left(1,{\hat{n}}_{\phi }\right)↔P_{\phi }\equiv \rho_{\phi }=\left(I_{2}+{\hat{n}}_{\phi }\cdot \sigma \right)/2$ (see Equation (6)), we can also write

(16) $$f_{\phi }\left[\left(r_{0},r\right)\right]=\mathrm{Tr}\left(\rho_{\phi }^{†}\rho_{\psi }\right).$$

In summary, under the above assumptions, $f_{\phi }\left(r\right)$ has reduced to be a scalar product. It can be specified either in vector space $V_{4}$, where it is given by the Euclidean scalar product, or in the space of linear operators acting on $H_{2}$, where it is given by the Hilbert–Schmidt inner product $\mathrm{Tr}(A^{†}B)$. Of course, $f_{\phi }\left(r\right)$ can be negative for some $r\in V_{4}$. However, if we restrict ourselves to applying $f_{\phi }\left(r\right)$ on vectors $\left(1,{\hat{n}}_{\psi }\right)\in V_{4}$, then $f_{\phi }\left[\left(1,{\hat{n}}_{\psi }\right)\right]\in \left[0,1\right]$, and in this case we may use $f_{\phi }$ as a probability measure. It is up to us to decide which mathematical tools we employ in order to describe our experimental observations. The probability measure $f_{\phi }$ is just one of these tools. As discussed in [11], it is not exclusively connected to quantum phenomena.

Let us now briefly refer to Hall’s criticisms [14] of our derivation of Born’s rule. Hall claims that our defining conditions for a measure $f_{\phi }$ are satisfied by the following non-linear measure:

(17) $$f_{\phi }\left(P_{\psi }\right)=\frac{1}{2}\left[1+f({\hat{n}}_{\phi }\cdot {\hat{n}}_{\psi })\right].$$

Here, f(x) “is any non-linear function mapping the interval [−1,1] into itself, with f(−x)=−f(x) and f(1)=1” [14]. The above $f_{\phi }$ can be proved to satisfy Gleason’s assumptions ((1) and (2)) in the 2D case, thereby showing that Gleason’s theorem does not hold for qubits. If f(x) is also required to be continuous, then $f_{\phi }$ should allegedly satisfy our defining conditions [14]. However, our function $f_{\phi }$ maps vectors in $V_{4}$ to the reals. For instance, these vectors may be of the form $\left(\pm 1,{\hat{n}}_{\psi }\right)$. On the other hand, the subject of the above definition, Equation (17), is a function whose domain is not $V_{4}$. Instead of Hall’s notation, $f_{\phi }\left(P_{\psi }\right)$, one should more properly write $f_{\phi }\left({\hat{n}}_{\psi }\right)$ on the lhs of Equation (17). The domain of Hall’s $f_{\phi }$ is thus the unit sphere. In particular, one cannot tell the results of applying this $f_{\phi }$ to vectors such as $\left(1,{\hat{n}}_{\psi }\right)$ and $\left(-1,{\hat{n}}_{\psi }\right)$. Hence, one cannot claim that this $f_{\phi }\left(P_{\psi }\right)$ satisfies, for example, the requirement given by Equation (14): $f_{\phi }\left[(-1,{\hat{n}}_{\phi })\right]=0$.

One can try to circumvent Hall’s technical flaw and still seek to object to our derivation of Born’s rule by arguing that qubits should not be treated as belonging to $V_{4}$. Such a claim connects with the belief that qubits are bijectively mapped to the points on the surface of the unit (Bloch/Poincaré) sphere, so that any given qubit |ψ〉 may be represented by some unit vector ${\hat{n}}_{\psi }$. This is wrong. Qubits (viz., spinors) span $V_{4}∼C^{2}∋|\psi \rangle =\alpha |↑\rangle +\beta |↓\rangle$, under variation of the complex-valued coefficients α and β. In order to restrict spinors |ψ〉 so as to span only the unit sphere $S^{2}:=\left\{\hat{n}\in R^{3}:\left|\hat{n}\right|=1\right\}\subset R^{3}$, we need to normalize |ψ〉 and discard a global phase. This amounts to neglecting some information that we deem unimportant, whatever the reason. However, under different circumstances, this information may turn out to be physically meaningful; see our closing remarks below, Section 5. An exhaustive description of qubits should therefore be given by the elements of $C^{2}∼V_{4}$.

The generalization of the above results to higher dimensional vector spaces and to mixed states is straightforward, and has been discussed in Ref. [11]. The generalization is based on the observation that two-dimensional Hilbert spaces are in fact general enough for dealing with the Born rule. Indeed, this rule involves only two states and therefore effectively limits itself—in each concrete case—to dealing with a two-dimensional subspace of the addressed vector space. This also holds in the case of infinite-dimensional spaces with continuous basis vectors |ϕ(α)〉, which may be thought of as eigenvectors of some observable with a continuous spectrum given by α. In such a case, one replaces the probability $f_{\phi }\left(P_{\psi }\right)$ in Born’s formula (13) by $df_{\phi (\alpha )}\left(P_{\psi }\right)={\left|\langle \phi \left(\alpha \right)|\psi \rangle \right|}^{2}d\alpha$, corresponding to measurement results between α and α+dα. Although this procedure leads to our intended goal, it is instructive to follow an alternative approach, in which we apply algebraic tools similar to those related to the Pauli algebra. This puts the qubit case on the same footing as the higher-dimensional ones. We present this approach next, restricted to systems of arbitrary finite dimension.

## 4. Gudder’s Theorem and the Born Rule for *N*-Level Systems

Let us first recall that the Pauli matrices are generators of the SU(2) group. Together with the 2×2 unit matrix, they constitute an orthonormal basis, in terms of which we can express any operator acting on the two-dimensional Hilbert space $H_{2}$. When dealing with higher dimensional spaces $H_{N}$, we can resort to the $N^{2}-1$ generators $G_{i}=G_{i}^{†}$ of the SU(N) group. These can be chosen so as to satisfy

(18) $$\mathrm{Tr}G_{i}=0,\;\;\mathrm{Tr}\left(G_{i}G_{j}\right)=N\delta_{ij}.$$

Notice that our choice of normalization is best suited to our present purposes and differs from the most commonly employed one, namely $\mathrm{Tr}\left(G_{i}G_{j}\right)=2\delta_{ij}$ [29,30,31,32]. Any operator $\rho =\rho^{†}$ with $\mathrm{Tr}\rho \equiv \sqrt{N}r_{0}$ can be expressed as

(19) $$\rho_{r}=\frac{1}{\sqrt{N}}\left(r_{0}I_{N}+\sum_{k=1}^{N^{2}-1}r_{k}G_{k}\right),$$

where $r_{k}\in R$, for $k=0,\ldots ,N^{2}-1$. This establishes a one-to-one correspondence between Hermitian operators ρ acting on $H_{N}$ and vectors $r\in V_{N}$. Let us now choose one of these vectors, $r_{\phi }=\left(r_{0},\ldots ,r_{d}\right)\in V_{N}$, where $d=N^{2}-1$. It corresponds to a fixed state $\rho_{\phi }$, a Hermitian operator that acts on $H_{N}$. We can represent the state $\rho_{\phi }$ in an alternative way, namely by means of Gudder’s measure $f_{\phi }$, the one that is the subject matter of Gudder’s Theorem 1. To begin with, we consider a vector $r_{⊥}$ orthogonal to $r_{\phi }$ (i.e., $r_{\phi }\cdot r_{⊥}=0$), and require that our measure yields a null result in this case: $f_{\phi }\left(r_{⊥}\right)=0$. The same requirement holds for vector $-r_{⊥}$, so that on view of Gudder’s theorem we have:

(20) $$f_{\phi }\left(r_{⊥}\right)=c_{\phi }r_{⊥}\cdot r_{⊥}+k_{\phi }\cdot r_{⊥}=0,$$

(21) $$f_{\phi }\left(-r_{⊥}\right)=c_{\phi }r_{⊥}\cdot r_{⊥}-k_{\phi }\cdot r_{⊥}=0.$$

The above requirements imply that $c_{\phi }=0$. Thus, Gudder’s measure $f_{\phi }$ reads $f_{\phi }\left(r\right)=k_{\phi }\cdot r$ in our case, with $k_{\phi }\in V_{N}$ yet to be determined. With $r_{\phi }$ and $d=N^{2}-1$ additional vectors $s_{\left(1\right)},\ldots ,s_{\left(d\right)}$, we can conform an orthogonal basis, in terms of which we can write $k_{\phi }=\lambda r_{\phi }+\sum_{j=1}^{d}\lambda_{j}s_{\left(j\right)}$. For the same reasons as before, we require that $f_{\phi }\left(s_{\left(j\right)}\right)=k_{\phi }\cdot s_{\left(j\right)}=0$ for j=1,…,d. This leads us to conclude that $k_{\phi }$ is parallel to $r_{\phi }$; i.e., $k_{\phi }=\lambda r_{\phi }$. If we finally require that $f_{\phi }\left(r_{\phi }\right)=1$, we end up with

(22) $$f_{\phi }\left(r\right)=\frac{1}{r_{\phi }\cdot r_{\phi }}r_{\phi }\cdot r.$$

By choosing the normalization $r_{\phi }\cdot r_{\phi }=1$, we have $f_{\phi }\left(r\right)=r_{\phi }\cdot r$. The normalization in Equation (19) has been chosen so as to render

(23) $$\mathrm{Tr}(\rho_{r}\rho_{s})=r\cdot s.$$

This allows us to write

(24) $$f_{\phi }\left(r\right)=r_{\phi }\cdot r=\mathrm{Tr}\left(\rho_{\phi }\rho_{r}\right).$$

It is a matter of convention which normalization we use; e.g., that of Equations (19) and (22), or else that of Equations (6) and (12). The Born rule is contained in Equation (24) when we restrict ourselves to suitably normalized vectors and operators. In that case, Gudder’s measure may be used as a probability measure. The general case corresponds instead to an inner product, which can be seen as a signed measure.

## 5. Closing Remarks and Discussion

According to Bohr, all quantum measurements require the involvement of a classical device. This assertion implies the unavoidable existence of two different domains—the classical and the quantal. That is, the quantum domain cannot be extended to embrace all physical phenomena, because these phenomena would include measurements themselves. Moreover, if we explicitly avoid dealing with the physical process that takes place during a measurement—that is, with possible changes suffered by a system when submitted to measurement—and focus on the quantification of the outcomes, then we cannot expect that this quantification has peculiar features that are exclusively ascribable to the quantum or to the classical domain. In other words, the Born rule by itself should equally well fit into a quantum and into a classical framework. The derivation of the Born rule presented here is in accordance with such a view. There is nothing in the framework we have used that can be identified as purely quantal. In particular, spinors—or their corresponding density matrices—are an appropriate and useful tool in both the quantum framework (e.g., spin-1/2 particles) and the classical framework (e.g., polarized light beams).

In order to obtain the Born rule, we drew upon Gudder’s theorem—a result that is tightly connected with a signed measure. By adding some requirements to the orthogonally-additive functions that are the subject matter of Gudder’s theorem, we got a twofold extension of Gleason’s theorem in which, first, qubits are included within the scope of the theorem and, second, Born’s probability rule arises as a special case of an inner product. Qubits may be understood as spanning a four-dimensional real vector space $V_{4}$ whose elements are of the form $\left(r_{0},r\right)$. The function *f* in Gudder’s theorem acts on this space, and is assumed to be continuous and orthogonally additive. When dealing with vectors of the particular form $\left(1,\hat{n}\right)$, we impose some additional requirements on *f*. These requirements let us interpret *f* as a probability measure $f_{\phi }$, which is defined in terms of some fixed state $\left(1,{\hat{n}}_{\phi }\right)$. When $f_{\phi }$ acts on more general vectors $\left(r_{0},r\right)$, then it acts as an inner product. As pointed out in Ref. [11], having discussed the two-dimensional Hilbert space, we have essentially discussed all higher-dimensional Hilbert spaces, at least with respect to Born’s rule. It is worthwhile to stress that the key requirements leading to the linearity of $f_{\phi }$ (i.e., $f_{\phi }\left(r\right)=k_{\phi }\cdot r$) are just two: $f_{\phi }\left(r_{⊥}\right)=0$ and $f_{\phi }\left(-r_{⊥}\right)=0$, cf. Equations (20) and (21). From them, it follows that c=0 in Theorem 1. Hence, as a consequence of these assumptions, $f_{\phi }$ turns out to be an odd function: $f_{\phi }\left(-r\right)=-f_{\phi }\left(r\right)$. Reciprocally, if $f_{\phi }$ is assumed to be odd, then it must be linear [12].

Concerning dimensionality, we should emphasize why we have dealt with $V_{4}$ in the case of qubits, instead of dealing with a space of lower dimensionality. Qubits are usually defined as normalized vectors in a two-dimensional Hilbert space, or equivalently, as projectors (i.e., density operators acting on this space). They can thus be represented as points on the 2D surface of a unit sphere that is embedded in 3D space. There are many ways in which one can embed a 2D surface in a higher-dimensional space. One can then ask about the physical motivation for dealing with $V_{4}$. Why do we not stay dealing with a 2D sphere? The physical motivation is given by mixed states in the case of spin-1/2 particles and by partially polarized light in the optical case. In these cases, we must deal with the whole Bloch ball and with the whole Poincaré ball, respectively, and not only with their surfaces. This is because the first component of a Poincaré or a Bloch vector $r\in V_{4}$ generally carries some physical information. For example, the intensity of polarized light is encoded in this first component. Although it might occur that we are not interested in knowing absolute but only relative intensity values and we consequently normalize our vectors, our formalism should nonetheless provide us with the option of accessing all the physical information that is connected with the phenomenon it is supposed to describe. This brings us outside the unit ball, and so we have to consider balls of arbitrary radii—the union of which makes up $V_{4}$. In the case of spin-1/2 particles, we naturally unit-normalize the density operator due to its interpretation in terms of probability. In that case, we usually do not need to go beyond the unit sphere. However, we could find it useful to connect probability with the actual number of particles we expect to detect in a given experiment. This could happen because of practical reasons, for example in order to avoid saturation of some detectors. In cases like this, we again need to go beyond the unit sphere in $V_{4}$. As an example of current theoretical interest, we may mention the study of qubits evolving according to quantum maps that are not completely positive, and therefore generally map the unit ball onto a set that is not contained in this ball [33]. The point in question seems to have been better appreciated by the classical community than by the quantum community, at least in the case of classical and quantum optics. Indeed, in classical optics one routinely uses either the Jones or the Mueller formalism. The latter deals with vectors in $V_{4}$, and perhaps no one would object that all four components of Mueller vectors have physical meaning. Some researchers even think that the Mueller formalism is more general and better suited than the Jones formalism to address physically-motivated inquiries [34]. Our approach acknowledges the fact that by dealing with 2D spinors some portion of physical information has been discarded. To take full account of this information, a 4D formalism is required, with the corresponding generalization in the SU(N) case.

Finally, we should emphasize that our goals substantially differ from Gleason’s. Indeed, we are not interested in showing that the structure of the Hilbert space naturally arises as the scenario in which quantum mechanics should be formulated. We have instead assumed that, say, qubits can be represented by density matrices in a Hilbert space, or else by four-dimensional vectors of a linear space. Our aim was to expose the fundamental underlying assumptions leading to a probability rule that has the structure of Born’s rule. By so doing, we can see the extent to which these assumptions lie in the quantum or in the classical domain. 
